# Triple reassortment increases compatibility among viral ribonucleoprotein genes of contemporary avian and human influenza A viruses

**DOI:** 10.1371/journal.ppat.1009962

**Published:** 2021-10-07

**Authors:** Kaitlyn Waters, Cheng Gao, Matthew Ykema, Lei Han, Lynden Voth, Yizhi Jane Tao, Xiu-Feng Wan

**Affiliations:** 1 Missouri University Center for Influenza and Emerging Infectious Diseases, University of Missouri, Columbia, Missouri, United States of America; 2 Department of Molecular Microbiology and Immunology, School of Medicine, University of Missouri, Columbia, Missouri, United States of America; 3 Bond Life Sciences Center, University of Missouri, Columbia, Missouri, United States of America; 4 Department of Basic Sciences, College of Veterinary Medicine, Mississippi State University, Starkville, Mississippi, United States of America; 5 Department of Electrical Engineering & Computer Science, College of Engineering, University of Missouri, Columbia, Missouri, United States of America; 6 Department of BioSciences, Rice University, Houston, Texas, United States of America; UNITED STATES

## Abstract

Compatibility among the influenza A virus (IAV) ribonucleoprotein (RNP) genes affects viral replication efficiency and can limit the emergence of novel reassortants, including those with potential pandemic risks. In this study, we determined the polymerase activities of 2,451 RNP reassortants among three seasonal and eight enzootic IAVs by using a minigenome assay. Results showed that the 2009 H1N1 RNP are more compatible with the tested enzootic RNP than seasonal H3N2 RNP and that triple reassortment increased such compatibility. The RNP reassortants among 2009 H1N1, canine H3N8, and avian H4N6 IAVs had the highest polymerase activities. Residues in the RNA binding motifs and the contact regions among RNP proteins affected polymerase activities. Our data indicates that compatibility among seasonal and enzootic RNPs are selective, and enzoosis of multiple strains in the animal-human interface can facilitate emergence of an RNP with increased replication efficiency in mammals, including humans.

## Author summary

In addition to humans, influenza A virus (IAV) infects avian, swine, canine, equine, and sea mammals. Genetic reassortment among two or more genetically diverse IAVs produce genetically distinct progeny virions and facilitated the emergence of at least three of the four documented pandemic IAVs in the past century. Compatibility among the IAV ribonucleoprotein (RNP) genes affects viral replication efficiency and can limit the emergence of novel reassortants, including those with potential pandemic risks. In this study, we evaluated the genetic compatibility among the RNP genes of human seasonal H1N1 and H3N2 IAVs and eight enzootic avian, swine, and caine IAVs at the animal human interface. Results showed that the 2009 H1N1 RNP are more compatible with enzootic RNPs than seasonal H3N2 RNP and that triple reassortment increases such compatibility. In addition, residues in the RNA binding motifs and the contact regions among RNP proteins affect polymerase activities of RNP reassortants. Our data indicates that genetic compatibility among avian and human RNPs are in general limited but not random, and the enzoosis of multiple strains in animal-human interactions can facilitate emergence of an RNP with increased replication efficiency in mammals, including humans. Decreasing the enzoosis and panzoosis of IAVs at the animal-human interface can help minimize the emerging risks of an IAV with a pandemic potential in humans.

## Introduction

Influenza A viruses (IAVs) are negative-sense, single stranded RNA viruses containing eight gene segments that encode at least 11 proteins. Based on antigenic properties of surface glycoproteins hemagglutinin (HA) and neuraminidase (NA), IAVs are grouped into 18 HA, and 11 NA subtypes [1–4]. In addition to humans, IAVs can infect a myriad of animal hosts including avian, swine, canine, equine, and sea mammals (e.g., seals and whales) [5–7]. IAVs of 16 HAs (H1-H16) and 9 NAs (N1-N9) have been recovered from wild aquatic birds, of which, migratory waterfowl and shorebirds, are the natural reservoir for IAVs [5].

The segmented nature of the influenza genome allows genetic assortment, which occurs when two or more genetically diverse viruses coinfect the same cell and exchange gene segments to produce genetically distinct progeny virions. Reassortment facilitated the emergence of at least three of the four documented pandemic IAVs. The 1957 H2N2 pandemic strain was a reassortant containing HA, NA, and polymerase basic 1 (PB1) of avian-origin and the other five genes (polymerase basic protein 2 [PB2], polymerase acidic protein [PA], nucleoprotein [NP], nonstructural [NS], and matrix [M]) genes from human seasonal H1N1 viruses [8]. The 1968 H3N2 pandemic strain had HA and PB1 of avian-origin and the other six genes from human seasonal H2N2 viruses [8,9]. The 2009 H1N1 pandemic virus contains genes of avian-origin (North American-lineage PB2 and PA), human-origin (PB1 from human seasonal H3N2), and swine origin (classic lineage HA and NP and Eurasian lineage NS, NA, and M) [10]. Thus, risk assessment of potential reassortants among epidemic IAVs and enzootic IAVs is considered a key component in pandemic influenza preparedness.

Replication and transcription of the IAV genome is performed in the nucleus of infected cells by the ribonucleoprotein (RNP) complex, which is formed by three polymerase proteins (PB2, PB1, and PA) in association with monomers of NP and viral RNA. Compatibility among RNP genes is an important factor restricting reassortment among IAVs. For example, equine H7N7 PB2 and PB1 combined with the human H3N2 PA gene were unable to form a heterotrimeric RNP complex [11]. At least three of four documented pandemic strains included RNP genes from both seasonal and enzootic IAVs. This indicates that the incorporation of one or more RNP genes from multiple hosts played a role in the reassortment events. Thus, understanding the compatibility of RNP genes from animal enzootic IAVs and human epidemic IAVs will facilitate assessment of reassortment risks among these IAVs.

This study presents an evaluation of the compatibility among the RNP genes between human seasonal and enzootic IAVs at the animal-human interface, and to further identify genetic features associated with RNP replication efficiency. In addition to human seasonal IAVs, we selected those IAVs frequently detected at the animal-human interface, especially those documented spillover infections from avian to human (i.e., subtypes H5N1, H7N9, H9N2), avian to canine (i.e., H3N2), avian to swine (i.e., H6N6 and H4N6), and equine to canine (i.e., H3N8). We determined the polymerase activities of 2,451 RNP reassortants among these viruses by using a minigenome assay, then applied a structure-guided sparse learning method to identify genetic features associated with polymerase activities.

## Results

### Genetic analysis of contemporary IAV RNP genes

In this study, we selected human seasonal H1N1 and H3N2 IAVs and eight enzootic viruses, which caused across species spillover cases (Table 1). Among these viruses, H5N1, H7N9, and H9N2 are of top priority for pandemic preparedness (https://www.who.int/influenza/preparedness/pandemic/en/). In particular, the A/goose/Guangdong/1/1996(H5N1)-like H5Nx IAVs were first detected in South China in 1996 [12,13]; in 2003, they spread to many countries across Asia, Europe, and Africa and, in 2014, they spread to North America[14]. These H5 IAVs have caused the loss of >300 million birds and 856 confirmed human cases, of which 53% were fatal. H4N6 IAVs are enzootic in the wild bird populations, and two spillovers of H4N6 were detected in domestic swine [15,16].

**Table 1:** List of contemporary IAVs at the animal-human interface used in this study.

Source	Abbreviation	Virus Name	IRAT risk score*
**Epidemic IAV**			Epidemic IAV
**Seasonal**	pdm09	A/California/07/2009(H1N1)	
**Seasonal**	swz13	A/Switzerland/9715293/2013(H3N2)	
**Seasonal**	mem94	A/Memphis/7/1994(H3N2)	
**Enzootic IAV**			Enzootic IAV
**Avian-origin**	H9N2	A/Hong Kong/308/2014(H9N2)	Moderate
**Avian-origin**	H5N1	A/Anhui/1/2005(H5N1)	Moderate
**Avian-origin**	H7N9	A/Guangdong/17SF003/2016(H7N9)	Moderate-High
**Equine-origin**	cH3N8	A/canine/Iowa/13628/2005(H3N8)	Low
**Avian-origin**	cH3N2	A/canine/Guangdong/1/2006(H3N2)	Low
**Avian-origin**	sH6N6	A/swine/Guangdong/K6/2010(H6N6)	Low
**Avian-origin**	sH4N6	A/swine/Missouri/A01727926/2015(H4N6)	Low
**Avian**	aH4N6	A/blue-winged teal/Ohio/12OS2244/2012(H4N6)	Low

*The Influenza Risk Assessment Tool (IRAT) was developed by the US Centers for Disease Control and Prevention (CDC) and WHO to assess the emergence risk and public health impact risk of a novel (i.e., new in humans) IAV [86,87]. The IRAT scores the risk by using 10 criteria based on biologic domain knowledge (e.g., virus properties, such as changes with known molecular signatures, receptor binding, transmission potential in laboratory animals, and drug susceptibility/resistance); population attributes (i.e., existing immunity, susceptibility to infection, severity of illness, and antigenic relationship to vaccine candidates); and virus ecology and epidemiology (i.e., global distribution, infections in animals, and infections in humans). IRAT scores the risks from 1–10: “low risk” is associated with a point score between 1 and 3; “moderate risk” between 4–7; and, “high risk” between 8–10.

To evaluate whether these genes cover the diversity of the IAV genetic pool in nature, we performed phylogenetic analyses of these genes along with those available in public databases. Results showed that there is a large diversity among each of these four RNP genes (S1 Fig). The genes from 11 tested viruses, in general, represent the diverse and predominant RNP lineages of contemporary IAVs (S1 Fig).

A total of 14,630 heterologous reassortant RNP combinations are possible among these 11 tested strains. To make this study more feasible, we focused on 51 triple RNP reassortant sets, each with 81 possible combinations (among one seasonal RNP and two enzootic RNP), resulting in a total of 2,451 reassortant RNP combinations (Table 1). These 51 RNP sets were designed to mimic those potential reassortants to emerge at the animal-human interface, including 1) combinations among human (pdm09, swz13, or mem94), canine (cH3N8 or cH3N2), and avian-origin swine (sH4N6 or sH6N6); 2) combinations among human (pdm09, swz13, or mem94), avian (aH4N6), and canine/swine (cH3N8, cH3N2, sH4N6 or sH6N6); 3) combinations among human (pdm09, swz13, or mem94), avian (aH5N1), and canine/swine (cH3N8, cH3N2, sH4N6 or sH6N6); 4) combinations among human (pdm09, swz13, or mem94), avian (aH7N9), and canine/swine (cH3N8, cH3N2, sH4N6 or sH6N6); and 5) combinations among human (pdm09, swz13, mem94), avian (aH4N6), and avian (aH9N2) (Table 2).

**Table 2:** A list of 51 triple reassortants among contemporary IAV RNP genes. These RNA combination were designed to mimic those potential reassortants to emerge at the animal-human interface.

Groups	Seasonal IAV	Enzootic IAVs		Median of Renilla/Firefly (R/F) (Upper/Lower)	RNP Reassortants^*a*^
1	pmd09	cH3N2	sH6N6	0.7458 (11.1982/0.0022)	33
	pmd09	cH3N2	sH4N6	2.4222 (23.3949/0.0074)	48
	pmd09	cH3N8	sH6N6	3.2170 (33.6905/0.0390)	58
	pmd09	cH3N8	sH4N6	4.4280 (33.6905/0.1293)	71
	swz13	cH3N2	sH6N6	0.3295 (17.9580/0.0061)	1
	swz13	cH3N2	sH4N6	0.9720 (23.2904/0.0150)	9
	swz13	cH3N8	sH6N6	1.3159 (33.6905/0.0076)	7
	swz13	cH3N8	sH4N6	3.1606 (33.6905/0.0455)	15
	mem94	cH3N2	sH6N6	0.3382(13.0152/0.0037)	12
	mem94	cH3N2	sH4N6	1.6304 (27.0439/0.0046)	22
	mem94	cH3N8	sH6N6	1.8277 (33.6905/0.0044)	27
	mem94	cH3N8	sH4N6	3.4923 (33.6905/0.1793)	32
2	pmd09	aH4N6	cH3N2	1.2640 (31.9297/0.0074)	42
	pmd09	aH4N6	cH3N8	4.5898 (38.1086/0.1193)	58
	pmd09	aH4N6	sH6N6	2.0265 (28.2910/0.0400)	48
	pmd09	aH4N6	sH4N6	4.2465 (25.9741/0.1193)	65
	swz13	aH4N6	cH3N2	2.0738 (33.5601/0.0062)	16
	swz13	aH4N6	cH3N8	3.5488 (33.6905/0.0796)	24
	swz13	aH4N6	sH6N6	2.5042 (34.5796/0.0156)	14
	swz13	aH4N6	sH4N6	2.5042 (33.5601/0.0176)	19
	mem94	aH4N6	cH3N2	1.8110 (24.4856/0.0065)	28
	mem94	aH4N6	cH3N8	3.5271 (33.6095/0.0393)	34
	mem94	aH4N6	sH6N6	2.3733 (28.2910/0.0026)	26
	mem94	aH4N6	sH4N6	3.8231 (22.1105/0.0258)	34
3	pmd09	aH5N1	cH3N2	0.5187 (11.1982/0.0074)	17
	pmd09	aH5N1	cH3N8	1.3568 (33.6905/0.0008)	46
	pmd09	aH5N1	sH6N6	0.0349 (7.5047/0.0004)	18
	pmd09	aH5N1	sH4N6	0.1709 (8.7817/0.0046)	25
	swz13	aH5N1	cH3N2	0.2607 (17.9580/0.0005)	1
	swz13	aH5N1	cH3N8	0.5441 (33.6905/0.0005)	4
	swz13	aH5N1	sH6N6	0.0501 (21.4277/0.0010)	1
	swz13	aH5N1	sH4N6	0.1884 (19.5517/0.0006)	4
	mem94	aH5N1	cH3N2	0.2514 (17.8895/0.0012)	5
	mem94	aH5N1	cH3N8	0.2800 (33.6905/0.0008)	10
	mem94	aH5N1	sH6N6	0.0229 (8.1934/0.0012)	6
	mem94	aH5N1	sH4N6	0.1467 (20.8613/0.0012)	9
4	pmd09	aH7N9	cH3N2	0.4794 (11.1982/0.0008)	24
	pmd09	aH7N9	cH3N8	1.3866 (33.6905/0.1523)	42
	pmd09	aH7N9	sH6N6	1.1405 (16.7195/0.0234)	39
	pmd09	aH7N9	sH4N6	0.7656 (8.7817/0.0921)	31
	swz13	aH7N9	cH3N2	0.7819 (22.9212/0.0025)	4
	swz13	aH7N9	cH3N8	1.4494 (33.6905/0.0986)	8
	swz13	aH7N9	sH6N6	0.7425 (22.8418/0.0023)	8
	swz13	aH7N9	sH4N6	0.9094 (19.5517/0.0380)	5
	mem94	aH7N9	cH3N2	0.4588 (20.3060/0.0025)	12
	mem94	aH7N9	cH3N8	2.0556 (33.6905/0.0133)	26
	mem94	aH7N9	sH6N6	0.8887 (20.3060/0.0016)	22
	mem94	aH7N9	sH4N6	0.8920 (20.8613/0.0125)	19
5	pdm09	aH9N2	aH4N6	0.4325 (12.2585/0.0098)	19
	swz13	aH9N2	aH4N6	0.3915 (33.5601/0.0025)	6
	mem94	aH9N2	aH4N6	0.6155 (23.8123/0.0024)	18

^***a***^Number of compatible RNP reassortants with polymerase activity greater than corresponding WT seasonal human RNP.

### Contemporary avian and canine RNP can increase polymerase activities of human RNP but the compatibility is mostly limited

In order to evaluate RNP compatibility, the polymerase activities of the wild-type (WT) RNP for the testing IAVs were quantified in HEK 293T cells at 37°C using a minigenome assay as described elsewhere [17]. Briefly, cells were transfected with four plasmids expressing PB2, PB1, PA, and NP, each with a human RNA pol I promoter and a human cytomegalovirus (HCMV) pol II promoter, a plasmid expressing *Renilla* luciferase with a human RNA pol I promoter, and another plasmid expressing *firefly* luciferase but with a simian virus 40 (SV40) pol II promoter. Through the HCMV pol II promoter, mRNAs for the four viral RNP genes are first syntheized by the host polymerase, and then translated to produce the four RNP proteins, which then functions to package, replicate and transcribe the negative-sense RNAs of the viral PB2, PB1, PA, and NP genes, as well as the Renilla luciferase reporter produced from the human RNA pol I promoter. The SV40 pol II promoter ensures that the *firefly* luciferase activity is transcribed and translated by host machinery independent of the viral RNP. During transfection, the same amount of viral RNP and Renilla luciferase plasmids are used while host cell numbers remain consistent. Therefore, a higher *Renilla* luciferase activity would signify a more efficient polymerase activity of testing RNP complex, whereas the *firefly* luciferase activity is expected to be constant. To make the analyses quantifiable, the luciferase activity for testing an RNP complex is calculated by *Renilla/firefly* (R/F) values.

**Fig 1:**
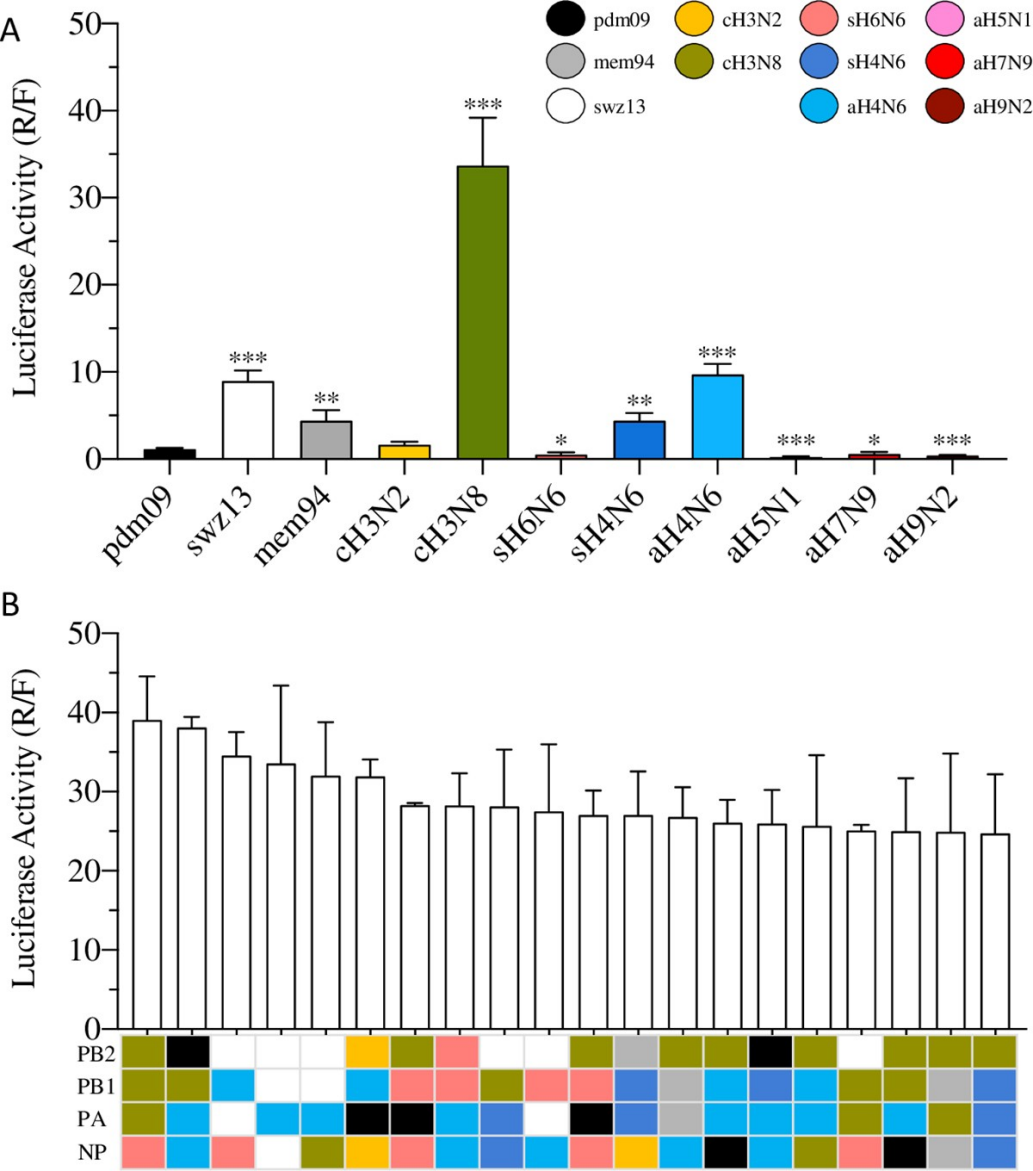
Polymerase activities of human seasonal and enzootic RNP complexes. **(A)** 293T cells were co-transfected with PB2, PB1, PA, and NP plasmids from WT pdm09, swz13, cH3N8, sH4N6, H5N1, sH6N6, H7N9, mem94, cH3N2, or aH4N6 IAV strains along with the *firefly* reporter vector pGL4.13 [*luc2*/SV40] and the *Renilla* luciferase expression plasmid. The values shown are ± from three independent experiments. * indicates *P* < 0.01, ** indicates *P* < 0.001, and *** indicates *P* < 0.0001 when compared to pdm09; **(B)** Polymerase activities of top 20 RNP reassortants.

Results showed a wide range of polymerase activities among the 11 WT RNP sets. The pdm09 RNP has a moderate R/F value of 1.15 (±0.11). Compared to the pdm09 RNP, human seasonal H3N2 RNP (swz13 and mem94), North America avian-origin viruses (sH4N6 and aH4N6), and equine-origin cH3N8 have significantly higher polymerase activities, whereas four Eurasian-origin avian RNPs (aH5N1, sH6N6, aH7N9, and aH9N2) had significantly lower polymerase activities (Fig 1A). The polymerase activity of avian-origin cH3N2 RNP was not statistically different from that of pdm09 RNP. Previous studies showed that all three of the most recent pandemic strains contained at least one seasonal RNP gene and others from enzootic IAVs at the animal-human interface [8–10]. Thus, by assuming that a reassortant RNP with a high polymerase activity would facilitate the emergence of zootic influenza viruses in humans, in the following analyses, the genes in RNP reassortants are defined as compatible if the polymerase activities are greater than those of the corresponding human seasonal WT (pdm09, swz13, or mem94) RNP, and, otherwise, as incompatible.

Results showed that there is a large variation in polymerase activities among the tested RNP sets. Of the 51 triple RNP reassortant test sets, a wide range (from 1 to 71) of RNP reassortants (out of 81 in total) had increased polymerase activities than the corresponding human seasonal WT RNP (Table 2, S1 and S2 Tables). Overall, the triple RNP reassortants among human seasonal, H4N6 (aH4N6 or sH4N6) and canine (cH3N2 or cH3N8) IAVs had the highest polymerase activities (Fig 1B). Of note, among the top 20 RNP reassortants with the highest polymerase activities, 18 contained at least one gene from cH3N8 or aH4N6 (Fig 1B). Interestingly, 16 of these 20 RNP reassortants are triple reassortants containing one gene from human seasonal IAVs.

**Fig 2:**
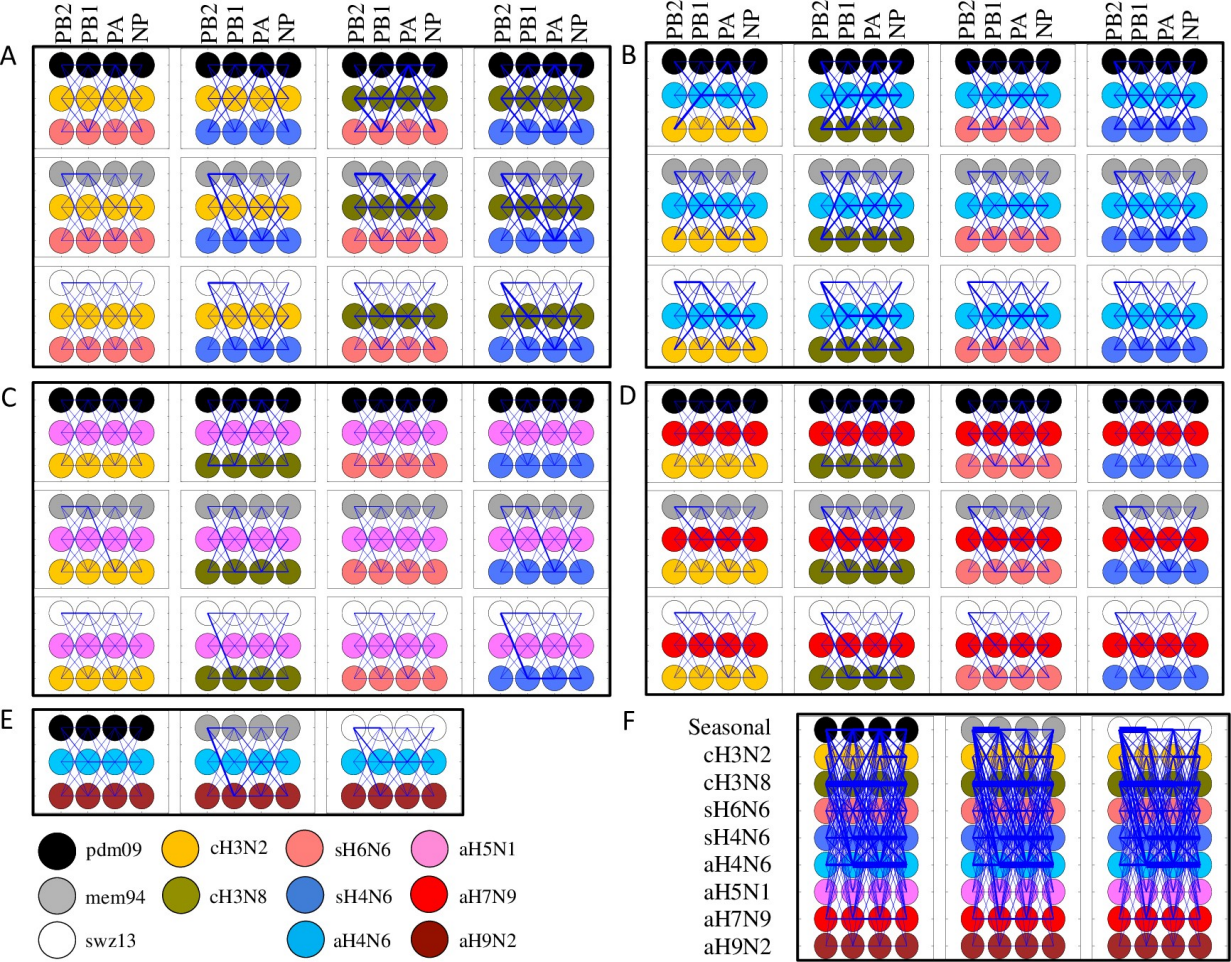
Representation of the 2,451 testing RNP reassortant complexes and the corresponding polymerase activities. **(A)** combinations among human (pdm09, swz13, or mem94), swine (sH4N6 and sH6N6), and canine (cH3N8 or cH3N2); **(B)** combinations among human (pdm09, swz13, or mem94), avian (aH4N6), and swine (sH4N6 and sH6N6) or canine (cH3N2 or cH3N8); **(C)** combinations among human (pdm09, swz13, or mem94), avian (H5N1), swine (sH4N6 and sH6N6) or canine (cH3N2 or cH3N8); **(D)** combinations among human (pdm09, swz13, or mem94), avian (H7N9), swine (sH4N6 and sH6N6) or canine (cH3N2 or cH3N8); **(E)** combinations among human (pdm09, swz13, or mem94), avian (H9N2), and (aH4N6); and **(F)** Contemporary IAV subtypes and corresponding host species used in this study are listed on the left-hand side, and PB2, PB1, PA, and NP genes from pdm09, swz13, and mem94 are represented by black, white, and gray circles, respectively.

Taken together, 2,451 tested RNP reassortant complexes revealed that compatibility is limited among human seasonal IAVs and most enzootic IAVs; human seasonal RNP genes are compatible the most with those tested enzootic RNP genes from contemporary North American IAVs (cH3N8, sH4N6, and aH4N6) (Fig 2).

### Triple reassortants can increase genetic compatibility among seasonal and enzootic IAV RNPs

**Fig 3:**
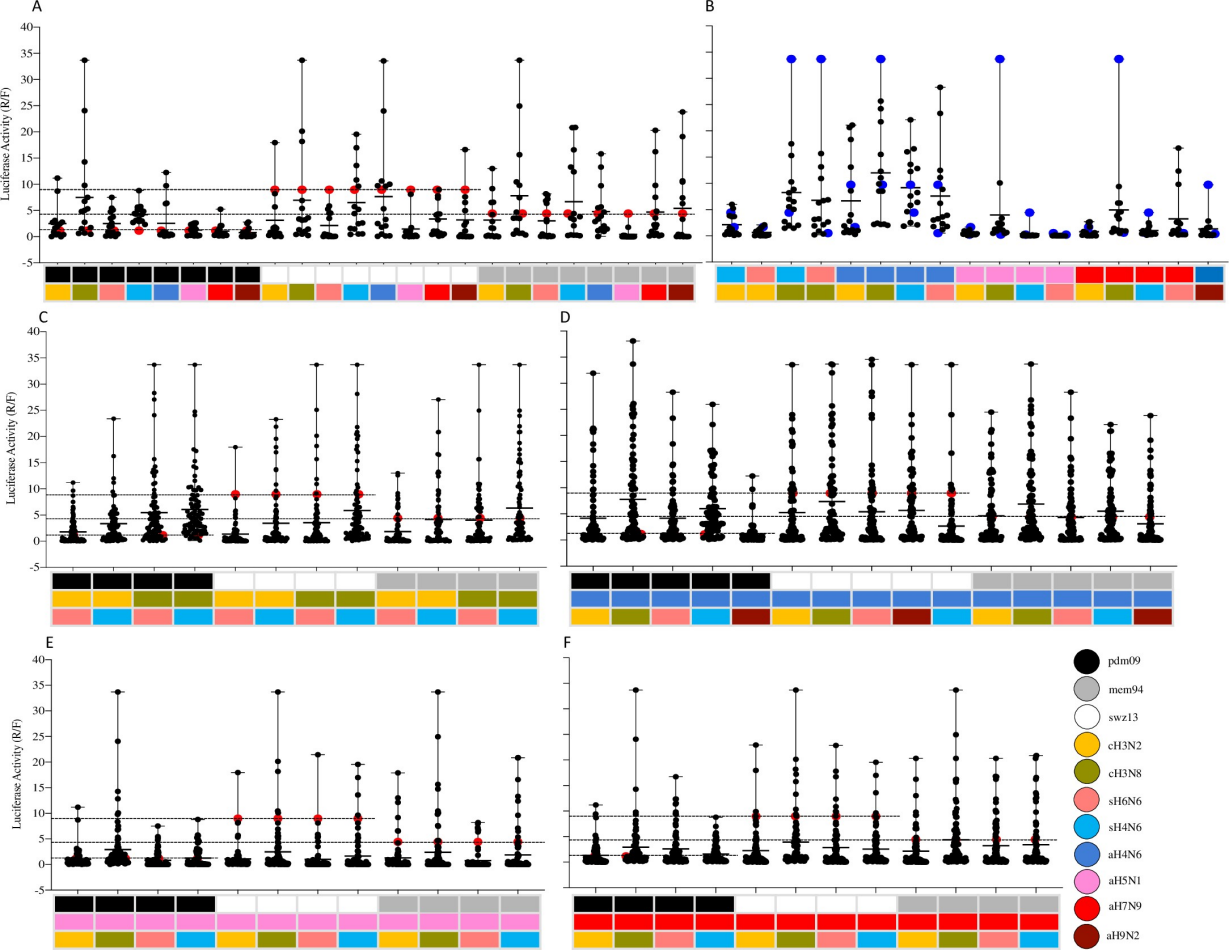
RNP reassortants with increased polymerase activities than the wild-type human seasonal or enzootic IAV RNP complexes. Representation of RNP reassortants with inceased polymerase than wild-type seasonal human RNP **(A)** among human-animal double RNP reassortants; **(B)** among mammalian-avian double RNP reassortants; **(C)** among human, swine, and canine triple reassortants; **(D)** among human, avian H4N6, and swine/canine triple reassortants; **(E)** human, avian H5N1, and swine/canine triple reassortants; **(F)** and among human, avian H7N9, and swine/canine triple reassortants. Each columan shows the polymerase activities for all of possible reassortants (n = 16 for double RNP reassortants (A-B), and n = 64 for triple RNP reassortants [C-E]) between two (A-B) or three RNP (C-E) sets, each of which was shown in color. The dash lines indicate the polymerase activities of the test three wild type human seasonal RNPs.

To understand genetic compatibility between a human seasonal RNP and an enzootic RNP, we analyzed the polymerase activities among RNP reassortants from 24 double seasonal-enzootic reassortant viruses (Fig 3A). Results showed that 112 out of 336 possible RNP double reassortants between human seasonal IAVs and testing enzootic IAVs had an increased polymerase activity compared to the corresponding human seasonal WT RNP (Fig 3A and S1 Table). Of interest, results of reassorment with an enzootic RNP showed that 61 RNP double reassorants with pdm09 had an increased compatibility whereas 15 and 36 RNP reassortants with swz13 and mem94, respectively, exhibited increased compatibility (Fig 3A and S1 Table). The median RNP reassortant polymerase activities of these reassortants were relatively higher for those RNP double reassortants with pdm09 than those with either swz13 or mem94 (S1 Table). On the other hand, we also compared genetic compatibility between 17 pairs of enzootic RNPs, and only 29 out of 238 possible RNP double reassortants exhibited an increased polymerase activity compared to both corresponding enzootic WT RNPs (Fig 3B and S2 Table).

Of interest, when we incorporated an RNP gene from a human seasonal IAV with those from any other two enzootic IAVs, both the median RNP reassortant polymerase activities and the percentage of the compatible RNP reassortants increased, especially when reassorted with pdm09 (Fig 3C–3F and Table 2). Particularly, 71 out of 78 possible RNP reassortants among pdm09, cH3N8, and sH4N6 yielded polymerase activities greater than the wild-type RNP of pdm09 (Table 2). Compared with those from aH4N6, the inclusion of aH5N1 or aH7N9 RNP genes were less likely to increase the polymerase activities. Conversely, compared with those from cH3N2, the incorporation of cH3N8 RNP genes is more likely to increase the polymerase activities when reassorting with those from human seasonal IAVs. Among two avian-origin swine viruses, more RNP reassortants with segments from sH4N6 exhibited higher polymerase activities than those with segments from sH6N6.

In summary, these results suggested that triple reassortment can increase genetic compatibility among human seasonal and enzootic IAV RNPs. Particularly, among all the tested RNP reassortant sets, those with canine H3N8 or swine H4N6 RNP are more likely to increase RNP activities when reassorted with pdm09, than those with canine H3N2 or swine H6N6, and similar results were observed among RNP reassortants from those enzootic IAVs with seasonal swz13 and mem94. Of note, genetic compatibility was reduced when RNP reassortment occurred between avian IAVs (aH5N1, aH7N9, or aH9N2) and human seasonal IAVs and between avian IAVs (H5N1, aH7N9, or aH9N2) and another enzootic IAV.

### Residues associated with polymerase activities including the contact residues between RNP proteins

To map residues affecting the polymerase activities, we naturally formulated this problem as a machine learning algorithm aiming to select feature residues (input variables) affecting polymerase activities (output variables). Specifically, we defined the luciferase activities as the phenotype output variables, and amino acid substitutions as input feature variables. A protein structure guided sparse learning algorithm, the generalized hierarchical sparse model (GHSM)[18], was used in this study. We compared the structure-guided GHSM with the L1-norm regularized method (LASSO) [19,20], the L1- and L2-norm regularized method (Elastic Net) [21], and the L1- and L∞-norm method (iCAP) [22] (see Supplementary Information [SI]). The 10-fold cross-validation shows that the structure-guided GHSM had the best performance (S3 Table); hence, we used structure-guided GHSM for our further analysis described below.

**Fig 4:**
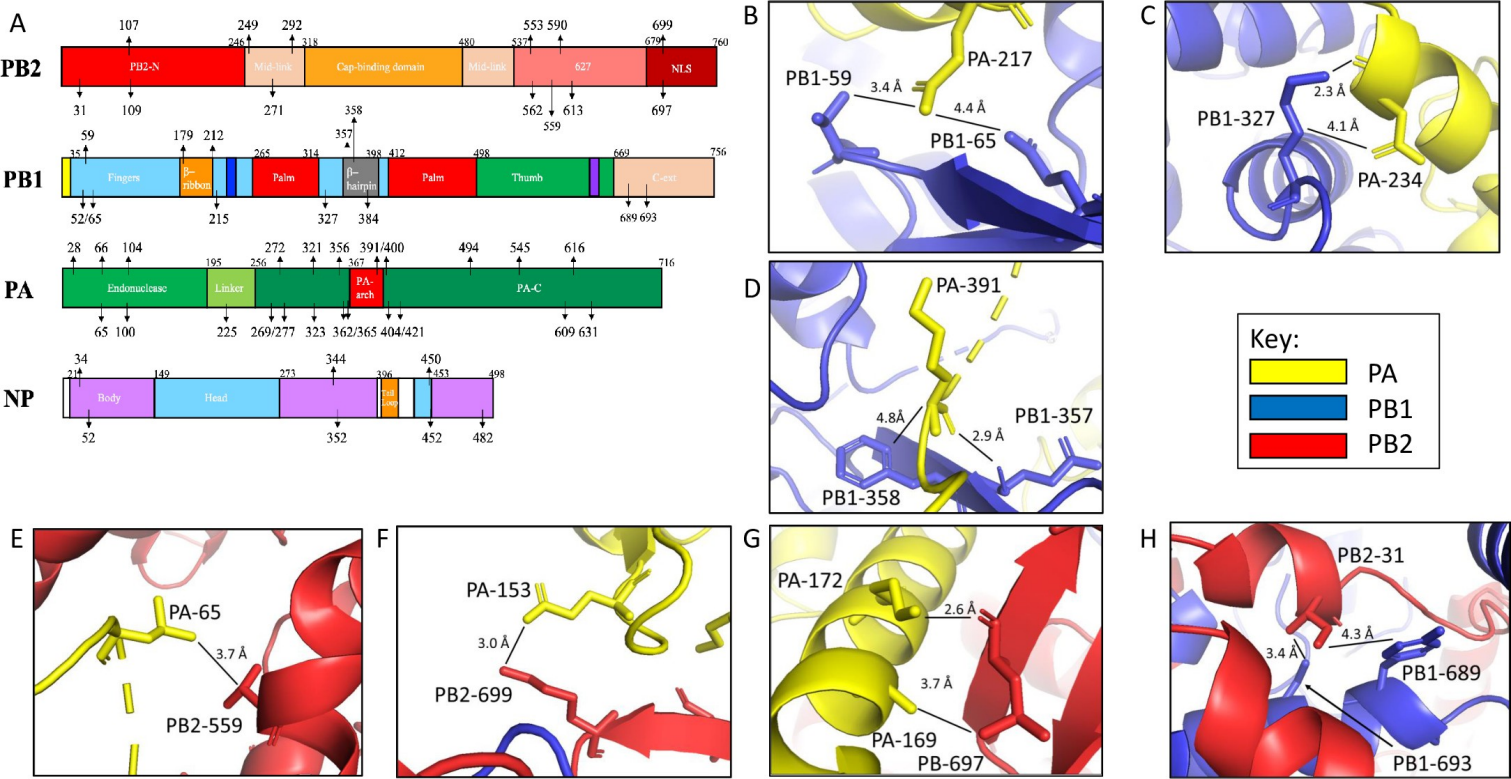
Schematic of RNP subunit domains with identified features mapped by structure guided machine learning. **(A)** PB2, PB1, PA, and NP subunit domain structures with subdomain names, and the residues identified by machine learning model as associated with polymerase activities are labeled. **(B-H)** RNP mutation sites found through machine learning were correlated with mutations that can influence polymerase activities (Table 3). From these mutations, 7 clusters of mutations were in locations that may influence interdomain interactions. Panels B-D models mutation clusters between PA and PB1. Panels E-G models mutation clusters between PA and PB2. Panel H models a cluster between PB1 and PB2.

A total of the top 53 features across the RNP complex were selected to be associated with polymerase activities (Table 3 and S4 Table). These residues are located in various functional domains of the RNP proteins that play vital roles in the transcription/replication of the viral genome, with 7 residues located at intersubunit interface making interactions with residues from another protein subunit, PB2-31|PB1-689/693, PB2-559|PA-65, PB2-697|PA-169/172, PB2-699|PA-153, PB1-59/65|PA-217, PB1-327|PA-234, PB1-357/358|PA-391 [23] (Fig 4 and Table 3).

**Table 3:** Residues in the ribonucleoprotein complex identified by machine learning to be associated with polymerase activities.

Feature ^a^	Diversity	Weight^*b*^	Comments
PA-400	A/L/P/Q	0.4294	
PA-404	A/S	0.3569	
PA-272	D/E/G/N	0.3162	
PB2-553	I/V	0.3210	RNA binding^[^^25^^]^
PB2-107	N/S	0.2516	interacts with NP^[^^24^^]^
PB1-215	K/R	0.2626	
**PA-391|PB1-357**	**K/R|F**	**0.2504**	**PA-391 directly contacts PB1-357** ^**[**^^**23**^^**]**^
**PA-391|PB1-358**	**K/R|E**	**0.2504**	**PA-391 directly contacts PB1- 58** ^**[**^^**23**^^**]**^
PB1-384	L/P/S	0.2424	binds cRNA^[^^32^^]^
PB1-179	I/M	0.2019	
**PA-65|PB2-559**	**L/S/Y|A/I/T**	**0.0214**	**PA-65 directly contacts PB2-559; endonuclease domain** ^**[**^ ^**88**^ ^**]**^
PB2-613	A/T/V	0.2050	RNA binding^[^^25^^]^; interacts with NP^[^^24^^]^; interacts with PB1^[^^24^^]^
PA-616	L/S	0.2157	interacts with PB1^[^^36^^,^^37^^]^
**PB1-59|PA-217**	**S/T|Q/Y**	**0.0779**	**PB1-59 directly contacts with PA-217; binds vRNA/cRNA**^**[**^^**32**^^,^ ^**89**^^**]**^
PB2-292	I/T/V	0.1553	
PB1-212	L/V	0.1586	
PA-269	K/R	0.1612	
PB1-52	K/R	0.1504	binds vRNA/cRNA^[^^32^^,^^88^^]^
PA-494	G/R	0.1677	interacts with PB1^[^^36^^,^^37^^]^
PA-101	D/E/G	0.0874	
NP-450	G/N/S	0.1447	NP oligomerization^[^^39^^]^; interacts with PB2^[^^38^^]^
PA-323	I/V	0.1280	
PB2-562	I/L	0.1412	RNA binding^[^^25^^]^
NP-34	D/E/G/S	0.1506	interacts with PB2^[^^38^^]^
PB2-249	E/G	0.1352	interacts with NP^[^^24^^]^
NP-482	N/S	0.1307	NP oligomerization^[^^39^^]^; interacts with PB2^[^^38^^]^
NP-352	M/V	0.1189	NP oligomerization^[^^39^^]^; interacts with PB2^[^^38^^]^
**PB1-327|PA-234**	**K/R|D/N**	**0.0164**	**PB1-327 directly contacts PA-234; binds cRNA** ^**[**^ ^**32**^ ^**]**^
PB2-271	A/T	0.09999	enhances polymerase activity in mammalian host cells^[^^30^^, 90]^
PA-356	K/R	0.0885	
NP-52	H/N/Y	0.0951	interacts with PB2^[^^38^^]^
PA-631	G/S	0.0990	interacts with PB1^[^^36^^,^^37^^]^
**PB2-31|PB1-689**	**I/V|Y**	**0.0928**	**PB2-31 directly contacts PB1-689; interacts with NP** ^**[**^ ^**24**^ ^**]**^
**PB2-31|PB1-693**	**I/V|C**	**0.0928**	**PB2-31 directly contacts PB1-693; interacts with NP** ^**[**^ ^**24**^ ^**]**^
**PB2-699|PA-153**	**K/R|E**	**0.0928**	**PB2-699 directly contacts PA- 153; RNA binding** ^**[**^ ^**25**^ ^**]**^ **; interacts with PB1** ^**[**^ ^**24**^ ^**]**^
PA-277	H/S/T/Y	0.0804	
NP-452	K/R	0.0980	NP oligomerization[39]; interacts with PB2^[^^38^^]^
PA-321	N/S/Y	0.0917	
PA-28	L/P	0.0856	endonuclease domain^[^^89^^]^
PA-66	D/G	0.0856	endonuclease domain^[^^89^^]^
PA-225	C/S	0.0856	NLS involvement^[^^33^^,^^34^^]^; host marker in pandemic and H5N1 IAVs^[91]^
NP-344	L/S	0.0965	NP oligomerization^[^^39^^]^; interacts with PB2^[^^38^^]^
**PB2-697|PA-169**	**I/L|A**	**0.0795**	**PB2-697 directly contacts PA-169; RNA binding** ^**[**^ ^**25**^ ^**]**^ **; interacts with PB1** ^**[**^ ^**24**^ ^**]**^
**PB2-697|PA-172**	**I/L|K**	**0.0795**	**PB2-697 directly contacts PA-172; RNA binding** ^**[**^ ^**25**^ ^**]**^ **; interacts with PB1** ^**[**^ ^**24**^ ^**]**^
PB2-590	G/S	0.0914	Compensates for lack of E627K in 2009 pandemic H1N1 viruses^[^^29^^,^ ^30^^]^; RNA binding^[^^25^^]^; interacts with NP^[^^24^^]^; interacts with PB1^[^^24^^]^
PA-100	A/V	0.0951	endonuclease domain^[^^89^^]^
PA-104	K/R	0.0839	endonuclease domain^[^^89^^]^
PA-545	I/V	0.0839	interacts with PB1^[^^36^^,^^37^^]^
PA-609	K/R	0.0839	interacts with PB1^[^^36^^,^^37^^]^
PA-365	H/Q/R	0.0820	
PA-421	I/S/V	0.0771	interacts with PB1^[^^36^^,^^37^^]^
PA-362	K/R	0.0948	
**PB1-65|PA-217**	**P/Q|Q/Y**	**0.0235**	**PB1-65 directly contacts PA-217; binds vRNA/cRNA**^**[**^^**32**^^,^ ^**89**^^**]**^

^*a*^The features were derived using structure-guided generalized hierarchical sparse model (GHSM), and the contact residues between the RNP proteins are set apart with a bold font

^*b*^The absolute value of each weight is derived from the GHSM model.

**Fig 5:**
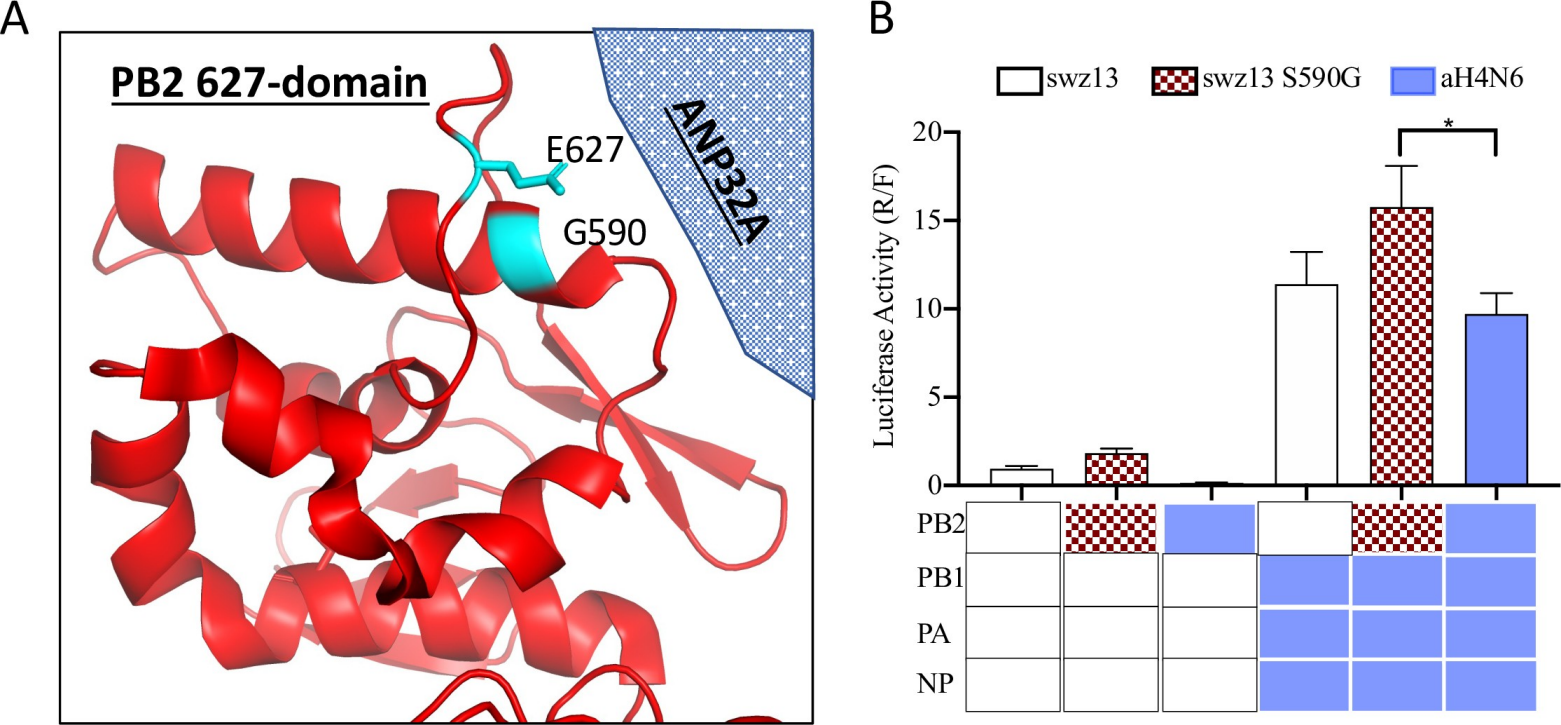
Polymerase activities of RNP reassortant complexes with a mutation at residue 590 of PB2 identified by machine learning. **(A)** The visualization of three dimensional structure of PB2 residue 590 (PDB ID 6QNW) in close proximity to host adaptation residue 627 was performed using PyMol (https://pymol.org). Only the 627 domain is shown in this view. Both residues 627 and 590 should be located at or near the interface with the host factor ANP32A according to the structure of the influenza C virus polymerase in complex with ANP32A. ANP32A is represented by the shaded object. **(B)** Polymerase activity of swz13 RNP complex with swz13-PB2 harboring S590G or wild-type aH4N6-PB2 and aH4N6 RNP complex with wild-type swz13-PB2 or swz13-PB2 harboring S590G. A/Switzerland/9715293/2013 (H3N2) is abbreviated as swz13 and A/blue-winged teal/Ohio/12OS2244/2012 (H4N6) as aH4N6. The values shown are ± from three independent experiments. *indicates *P*< 0.05 when compared to aH4N6.

Of these selected features, in addition to those direct contact residues discussed above, PB2-31, -107, -109, and -249 are in a region of the protein that contacts NP [24]. PB2-553, -562, -590, and -613 are located in the 627-domain, which has been postulated to be involved in RNA binding [25] and essential for the accumulation of cRNA replication intermediate in infected cells [26]. In particular, PB2-627 has been found to play a critical role in host adaption by mediating polymerase interaction with the host protein ANP32A to facilicate polymerase dimer assembly [27,28]. PB2-590 directly contacts PB2-627 (Fig 5A), and it has been reported that PB2-590 was able to compensate for the lack of E627K in 2009 pandemic H1N1 viruses [29,30]. Lastly, PB2-109, -271 and -292 are located in the Mid-link region that is implicated in bridging the 627-domain with PB2 cap binding domain and the PA subunit.

**Fig 6:**
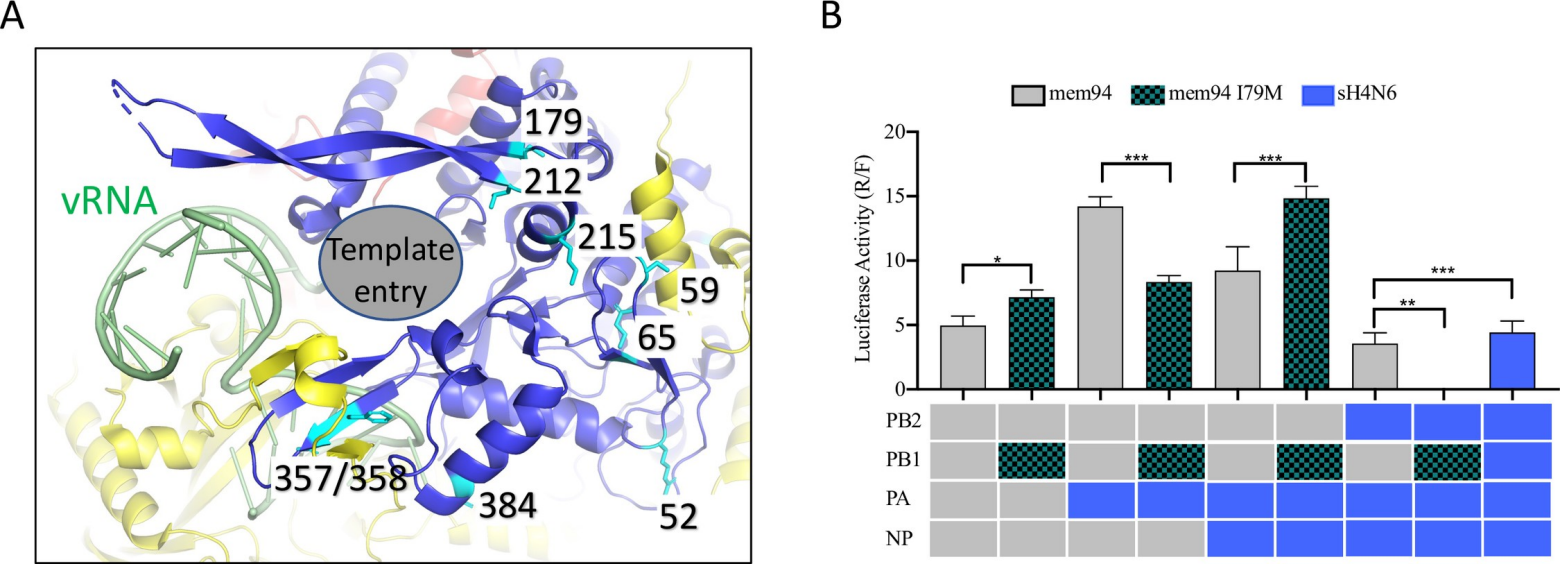
Polymerase activities of RNP reassortant complexes with a mutation at residue 179 of PB1 identified by machine learning. **(A)** The visualization of three dimensional structure of PB1 (PDB ID 4WSB) with most of the featured residues mapped near the template entry channel (grey oval) and the vRNA binding site. In particular, M179 is located at the base of an extended beta-hairpin that directly contact vRNA and possibly RNA template as well. PA: yellow; PB1, blue; PB2, red; vRNA, green. **(B)** Polymerase activity of mem94 RNP complex with mem94-PB1 harboring I179M and sH4N6 RNP complex with wild-type mem94-PB1 or mem94-PB1 harboring I179M. A/Memphis/7/1994 (H3N2) is abbreviated as mem94 and A/swine/Missouri/A01727926/2015 (H4N6) as sH4N6. The values shown are ± from three independent experiments. *indicates *P* < 0.05 and *** indicates *P* < 0.01 when compared to mem94.

Among features associated with PB1, PB1-52, -59, and -65 are located in a β hairpin that lies at the outer edge of the RNA template entry channel next to the PA-linker domain. Also found in an adjacent area are PB1-179, -212 and -215 at the base of an extended β hairpin that directly bind the c/v-RNA promotor and possibly the RNA template. The three latter residues are also adjacent to the two nuclear localization signals (NLS) that are important for binding the PB1-PA heterodimer nuclear import factor, RanBP5 (Fig 6A)[31]. Interestingly, PB1-384 is also in a nearby region surrounding the RNA template entry channel [32].

Among the four RNP proteins, PA has the most with 23 features in which 16 of these residues are located in the large PA-C domain and five in the endonuclease domain, with one residue located in both the PA-linker and -arch domains (Fig 4). It has been reported that PA-225 is in a region of the protein involved in nuclear localization [33,34]. PA-356, -362, and -365 are adjacent to the bound vRNA promotor [35]. In addition, PA-421, -494, -545, -609, -616, and -631 contact PB1 [36,37].

Finally, NP-34, -52, -344, -352, and -482 are located in the body domain, while NP-450 and -452 are in the head domain of NP. Both the body and head domains of NP interact with either PB2 or PB1 [38]. All seven of these residues are in NP regions that contact PB2 [38], while NP-344, -352, -450, -452, and -482 are in a region that is important for NP oligomerization [39].

In summary, the residues associated with RNP polymerase activities are found in functional regions of polymerase and NP proteins that are integral for transcription/replication of the IAV genome. They are also in contact regions among RNP proteins.

### Polymerase activity of RNP reassortants associated with features in PB2 and PB1 identified by machine learning in various influenza RNP complex backgrounds

To validate the roles of the selected residues on the RNP function, two residues, PB2-590 and PB1-179, were selected for site-directed mutagenesis followed by polymerase activity analyses by using the minigenome assay. As described above, PB2-590 is in close proximity to the PB2-627 host adaptation residue (Fig 5A). PB-1179 is located at the base of an extended beta-hairpin that directly contact vRNA and possibly RNA template, and PB1-179 is grouped in the same area with PB1-52, PB1-168, and PB1-212 (Table 3) near the PB1:PA subunit interface and could form a inter-subunit connection with the PA gene (Fig 6A). PB2-590 is in the human seasonal pdm09/swz13 and in the canine cH3N8 encode serine, but those in mem94 and all other enzootic viruses harbor glycine (Table 3). PB1-179 encodes isoleucine in human seasonal pdm09 and mem94, but methionine is encoded in swz13 and all of our enzootic viruses (Table 3). The swz13 PB2 was used as a template with PB2-590S or -590G, and the mem94 PB1 was used as the template with PB1-179I or 179M.

Results showed that compared to PB2-590S, PB2-590G increases polymerase activity, but not significantly (Fig 5B). To further understand the impact this mutation has on a divergent influenza RNP background, we introduced the swz13 PB2 into the aH4N6 RNP background. We found that swz PB2 slightly increased polymerase activity compared to the wild-type aH4N6 RNP complex. The addition of the swz13 S50G PB2 mutant to the aH4N6 RNP background further increases polymerase activity (Fig 6B).

With the mem94 PB2, PA, and NP, the I179M mutation significantly increases polymerase activity (Fig 6B). However, our results showed that the impacts of this mutation on polymerase activities are dependent on the genetic background of three other genes. With mem94 PB2, PB1, and NP, as well as sH4N6 PA, this mutation can lead to significant decreases in polymerase activities, similar to the situation with sH4N6 PB2, PA and NP. Notably, the I179M mutation substantially increased polymerase activities with mem94 PB2 and PB1, along with sH4N6 PA and NP.

Taken together, two selected residues, PB2-590 and PB1-179, were shown to affect RNP polymerase activities to different extents, but the effects may depend on the context of other proteins in the RNP complex.

## Discussion

Conventional methods for assessing the emergence risk of a novel IAV often require laboratory creation of reassortants and subsequent measurement of their infectivity, pathogenesis, and transmission in a mammalian system [11,40–51]. However, such a strategy is not only expensive and labor-intensive, but could also lead to artificial mutants with gain of function properties. An efficient strategy is needed to assess reassortment risks among enzootic IAVs in animals and human epidemic IAVs. Compatibility of IAV RNP is well documented to affect genetic reassortment among IAVs [11,52] and correlate with the replication/transcription efficiency, as well as the viral replication kinetics of reassortant viruses [11,40–43, 53]. For example, the 2009 H1N1 PA was shown to play a role in generating the reassortant viruses between the 2009 H1N1 pandemic and avian H9N2 strains [40]. RNP reassortment affects viral replication kinetics in swine respiratory tract cells of avian-origin H4N6 IAVs, and a virus with high replication ability in swine nasal cell is more likely to cause transmission in pigs [54]. To assess the emerging risk of novel zoonotic viruses to humans, we evaluated the compatibility among RNPs from human seasonal IAVs and those from eight enzootic IAVs at the animal-human interface, including those that have caused spillover events to human or mammals (i.e. dogs and pigs).

Among the 2,451 testing RNP reassortants, most did not exhibit a significant increase in RNP polymerase activities, suggesting that the compatibility of RNPs is, in general, selective. On the other hand, reassortment among RNP genes of IAVs does not occur randomly. For example, we found that the reassortment of pdm09 with sH4N6 (an avian-origin swine IAV that spilled over into pigs on a Missouri farm [15]) and cH3N8 (an equine-origin IAV) RNPs, both of which are contemporary North American IAVs, have increased RNP polymerase activity compared to the reassortants with sH6N6 (an avian-origin swine IAV that emerged in Asia [55] with potential public health risks [56]) and cH3N2, both of which are contemporary avian-origin Eurasian IAVs (Tables 1 and 2). Furthermore, incorporation of the aH4N6 RNP, a contemporary North American avian IAV, with those from pdm09 and contemporary North American IAVs (sH4N6 and cH3N8) or contemporary Eurasian IAVs (sH6N6 and cH3N2) increases RNP polymerase activity. These findings highlight the potential for RNP reassortment among human seasonal and enzootic IAVs, especially those enzootic North American IAVs. This potential RNP reassortment verifies the need for continued surveillance.

The triple reassortment internal gene (TRIG) cassette contains NP, NS, and M from swine, PB1 from humans, and PB2 and PA from avians [10]. Further, TRIG has been present in swine IAVs since the 1990s and facilitated the emergence of the 2009 H1N1 pandemic virus [10], emphasizing the important role of triple reassortment in the evolution of pandemic influenza viruses. In this research, we found that, compared with double reassortants, triple reassortants from human seasonal and enzootic IAVs produce more reassortants with RNP polymerase activities greater than the corresponding WT human seasonal RNPs (Fig 3 and Table 2); these results indicated that triple reassortment can increase genetic compatibility among human seasonal and enzootic IAV RNPs. Overall, our results suggested that the RNP of pmd09 is more compatible with our tested enzootic RNPs than the H3N2 seasonal RNPs, swz13 or mem94. For example, 33 triple RNP reassortants from pdm09, cH3N8, and sH4N6 had increased polymerase activities over the WT pdm09 RNP, whereas, 5 and 12 triple RNP reassortants from swz13 or mem94 with cH3N8 and sH4N6 had increased polymerase activities over the WT swz13 or mem94 RNPs, respectively (Fig 3C). Additionally, we found more triple RNP reassortants had greater polymerase activities containing pdm09 genes with aH4N6 (Fig 3D), aH5N1 (Fig 3E), and aH7N9 (Fig 3F) than those with either swz13 or mem94. The emergence and establishment of the 2009 pandemic H1N1 lineage in the human and swine population created a prime opportunity for reassortment between contemporary and seasonal influenza viruses that are co-circulating throughout the population [49]. During the past 10 years, with the rapid spread of the virus within global swine populations, the 2009 H1N1 viruses have substantially enhanced the diversity of the swine IAV genetic pool. A number of novel swine reassortants have been documented across North America, South America, Asia, and Europe. Among them, a novel avian-like G4-lineage H1N1 swine IAV was recently detected in China with evidence that it has become the predominant swine IAV [57]. Risk assessments have shown that this virus is highly transmissible among ferrets, and serosurveillance has shown that 10.4% of swine workers have been exposed to this novel virus. [57] In North America, at least nine genetic reassortants derived from 2009 H1N1 viruses were detected in domestic swine within a single year [58], and an H3N2 variant (H3N2v), containing a A(H1N1)pdm09-like matrix gene, was frequently detected in both domestic [59] and feral swine [60]. From 2011 to 2012, H3N2v viruses were estimated to have caused 2,055 humans infections [61]. Thus, the continuous emergence of novel swine IAVs with genetic elements derived from A(H1N1)pdm09, especially those with other IAVs at the animal-human interface, shall be a cause for great public health concern.

Mutations occurring in RNP genes have been identified as key determinants for host adaptation of avian IAVs to humans and other mammals, such as PB2 E627K [62–65]. However, post-reassortment adaptive mutations are most likely necessary to achieve an overall viral fitness that allows for successful reassortment [66]. For example, the 2009 pandemic H1N1 PB2 retained the avian-like E627 signature; however, the SR polymorphism, which denotes serine at position 590 and arginine at position 591, compensates for the lack of the human-like K627 signature [29]. Our machine learning model identified PB2-590 as a key feature among the 11 contemporary IAVs used in this study (Table 3). Recently, the mutation PA-K356R and its impact on host tropisms and the pathogenicity of avian H9N2, H7N9, and H10N8 IAVs have been investigated [67]. The K356R mutation emerged in avian H9N2 PA genes; however, due to reassortment events, it has since been incorporated into avian H7N9 and H10N8 IAV PA genes. The H9N2 PA R356 increased polymerase activities and viral replication kinetics in mammalian cells. This virus also caused severe lung pathology in mice [67]. PA-R356 is conserved among human 2009 pandemic H1N1, seasonal H1N1, and seasonal H3N2 strains, indicating that this mutation could facilitate the reassortment of avian H9N2 viruses in humans. Interestingly, PA-356 was identified by our machine learning model as a key feature (Table 3), and our pdm09 (H1N1), swz13 (H3N2), mem94 (H3N2), aH7N9, and aH9N2 strains each harbor R356 while the remaining enzootic strains have K356.

This study has several limitations. First, our analysis incorporates only appoximately 17% of all 14,630 possible RNP combinations among the 11 tested IAV. Further analysis to include some of the remaining 12,179 possible RNP reassortants could increase a more complete understanding RNP compitbility among these viruses. Secondly, our analyses were performed *in vitro*, particularly on the human HEK 293T cells. However, the experiments in this study may not completely reassemble the *in vivo* settings. On the other hand, the reassortment patterns in avian and swine cells might may not follow the same reassortment patterns in human cells. In addition, the emerging risks in this study was limited to only the RNP complex but not all eight genetic segments. To fully understand the emerging risks for co-circulating IAVs at the animal-human interface, future studies are needed to study the compability of the RNP reassortants from this study (e.g. by reverse genetics) with the other gene segments (HA, NA, NS, and MP) of the IAVs at the animal-human interface and futher to examine their infectivity, pathogenesis, and transmissionability using animal models.

In this study, we provide a large scale phenotypic analysis of reassortant RNP complexes from 11 contemporary human, avian, swine, and canine IAVs. Results showed that the 2009 H1N1 RNP are more compatible with enzootic RNPs than seasonal H3N2 RNP and that triple reassortment increases such compatibility. Residues in the RNA binding motifs and the contact regions among RNP proteins affect polymerase activities of RNP reassortants. Our data indicates that genetic compatibility among avian and human RNPs are in general limited but not random, and the enzoosis of multiple strains in animal-human interactions can facilitate emergence of an RNP with increased replication efficiency in mammals, including humans.

## Materials and methods

### Genomic Sequence, sequence alignment and phylogenetic analysis

The PB2 (22,240 sequences), PB1 (18,679 sequences), PA (22,576 sequences), and NP (12,762 sequences) protein sequences of all influenza virus hosts and subtypes were downloaded from the Influenza Research Database (https://www.fludb.org). Among them, 3,933 IAVs covered the full length of four RNP gene segments. Sequence alignments of each RNP segment were generated using Muscle v3.8.3. [68] Phylogenic analyses and bootstrap analyses were performed by using RAxML v8. [69] Phylogenetic trees were visualized by using FigTree v1.4.4.

To make phylogenetic tree construction feasible, we selected a small set of genes from the large data we downloaded. We applied a complete composition vector (CCV) [70] method to compute pairwise distances for each of the four gene segments, and used multidimensional scaling technique to projectevery sequence into a two dimensional coordiante system. We leveraged a hierarchical clustering method to cluster these sequences into different clusters. Hierarchical clustering starts by treating each observation as a separate cluster. Then, it repeatedly executes the following two steps: (1) identify the two clusters that are closest together, and (2) merge the two most similar clusters. This iterative process continues until all the clusters are merged together. We use the single linkage as our constraint, which treats the closest point distance of two clusters as the cluster distance. Single-linkage (nearest neighbor) is the shortestdistance between a pair of observations in two clusters. In single linkage hierarchical clustering, the distance between two clusters is defined as the shortest distance between two points in each cluster. For example, the distance between clusters “r” and “s” is equal to the length of the two closest points. *L*(*r*,*s*) = min (*D*(*x*_*ri*_,*x*_*sj*_)). After setting the threshold as 1, we obtained 15 clusters for PB2, eight clusters for PB2, 23 clusters for PA, and 18 clusters for NP. In order to construct phylogenetic tress, we randomly selected five data points of each cluster which includes more than 5 data points and remained the cluster with data points less than 5 for the four segments; to make the analyses be robust. We combined unique segments for three individual trails. The unique viruses selected out from the three trails were 161, 162 and 159 respectively.

### Cells and viruses

Madin-Darby Canine Kidney (MDCK) (NBL-3) CCL-34 and human embryonic kidney (HEK) 293T CRL-11268 cells (both from American Type Culture Collection, Manassas, VA) were incubated at 37°C with 5% CO_2_ and in Dulbecco’s Modified Eagle Medium (DMEM; GIBCO/BRL, Grand Island, NY), supplemented with 10% fetal bovine serum (Atlanta Biologicals, Lawrenceville, GA) and 1% penicillin–streptomycin and amphotericin B (GIBCO/BRL). The viruses used in this study are listed in Table 1.

### Reverse transcriptase-PCR (RT-PCR), molecular cloning, and site-directed mutagenesis

The full length PB2, PB1, PA, and NP genes from H5N1, H7N9, and H9N2 were synthesized by Gene Universal Inc (Newark, DE), and those from the other eight viruses were amplified using reverse transcriptase PCR. Specifically, viral RNA was isolated using GeneJET Viral RNA Purification Kit according to the manufacturer’s instruction (Thermo Fisher Scientific, Pittsburgh, PA). The reverse transcription was performed using SuperScript III Reverse Transcriptase (Thermo Fisher Scientific, Pittsburg, PA) and a pair of influenza A virus-specific primer Uni12 were used to amplify the full length gene fragments using the following PCR protocol: one cycle at 98°C for 30 sec, one cycle at 98°C for 10 sec, followed by 35 cycles at 53°C for 30 sec, 72°C for 2 min, and 72°C for 10 min. PCR products were then purified by gel electrophoresis and extracted using the GeneJET Gel Extraction Kit (Thermo Fisher Scientific, Pittsburgh, PA). Sanger sequencing services were performed at the University of Missouri DNA Core to confirm unexpected mutations were not introduced into the clones. These genes were then cloned into a pHW2000 vector [71] kindly provided by Dr. Richard Webby from St. Jude’s Children Research Hospital.

To validate the effects residues identified by machine learning have on RNP polymerase activities, we performed site-directed mutagensesis for the target gene, followed by minigenome analyses. Specifically, the QuickChange Lightning Site-Directed Mutagenesis Kit (Agilent Technologies, Santa Clara, CA, USA) was used to introduce mutations at residues of the PB2 protein from A/Switzerland/9715293/2013 (H3N2) and the PB1 protein from A/Memphis/7/1994 (H3N2). In total, one mutation was introduced into each protein. We used forward primer 5’-GACAAACCCACTGTATTGCCCTCTAATGGCCTTGGGGAC-3’ and reverse primer 5’-GTCCCCAAGGCCATTAGAGGGCAATACAGTGGGTTTGTC-3’ to generate mutation S590G into the A/Switzerland/9715293/2013 PB2. The forward primer 5’-TTGAAAGTGTGTTGTTATCTCCATTTCCTCTTTATCCATTGATTCC-3’ and reverse primer 5’-GGAATCAATGGATAAAGAGGAAATGGAGATAACAACACACTTTCAA-3’ was used to introduce the I179M mutation in the A/Memphis/7/1994 PB1. To ensure the absence of unwanted mutations, Sanger sequencing was performed by the DNA Core at the University of Missouri.

### Minigenome assay

The minigenome assay for a list of 51 triple reassortants among contemporary IAV RNP genes (Table 1, S1 and S2 Tables) was performed to evaluate the polymerase activities of RNP in HEK 293T cells at 37°C as described elsewhere (details in SI) [17]. Briefly, this assay started with seeding 4 ×10^4^ 293T in each well of a 96-well plate and transfecting them with 40 ng of 4 plasmids, each expressing one RNP gene (PB2, PB1, PA or NP), 40 ng of plasmid phPOLI*-*RLUC expressing *Renilla* luciferase, and 4 ng of pGL4.13 [luc2/SV40] expressing *firefly* luciferase (Promega, Madison, WI). Luciferase activities were measured in lysates from cells harvested at 48 h after transfection using the Dual-Luciferase Reporter Assay System (Promega, Madison, WI) according to manufacturer’s instructions. The ratio of *Renilla*/*firefly* luciferase activities was determined to measure the replication efficiency of a set of PB2, PB1, PA, and NP. The higher the ratio of *Renilla*/*firefly*, the higher the polymerase activities for the test RNP set. Each RNP combination was performed in triplicate. The mean of these data sets was used as the final value. The luciferase activities of WT and RNP reassortants were expressed as the ratio of *Renilla*/*firefly* (R/F) values and normalized to *Renilla* luciferase, which acted as the internal control. Thus, in each 96-well plate, one WT RNP was used as a positive control, and the activities of co-transfected *Renilla* and *firely* plasmid served as our negative control.

### Structure-guided sparse learning model

Sparse learning methods are advantageous for selecting a small number of important non-zero features from large amount of ones [72]. When data is limited, promoting sparsity has been shown to produce robust models that generalize well to extrapolated data [73]. It is well documented that only a small set of amino acids in influenza proteins are associated with changes in each of viral phenotypes such as antigenicity [74–76], receptor binding [77,78], replication [79–81], pathogenesis [82], and transmission [83], and such a biological setting enables sparse learning to be an ideal machine learning method to extract the features associated with these phenotypes for influenza. On the other hand, the data for phenotypic analyses suffers from a relatively small data size and high noise levels. Thus, sparse learning will be suitable in these problems. Previously, sparse learning has been successfully in key sequence features associated with antigenicity [20,84,85]. In this study, we developed a structure-guided sparse learning method to identify synergenetic residues across four RNP proteins affecting RNP polymeras activities (i.e., *Renilla/firefly* [R/F) values in the minigenome assay).

A total of 600 pairs were identified to be at the structural interfaces of PB2, PB1, PA, and NP proteins, 88 of which were with at least one mutation between contact amino acids among the viruses we used in this study. These paired contact residues were integrated with other individual residues to form the feature vector. To identify synergistic features in the feature vector, we adapted generalized hierarchical sparse model (GHSM) [18], a hierarchical sparse model to identify the potential combination of these features associated with the phenotypic changes in this study. The GHSM model aims to minimize: $L(W)+\sum_{k=1}^{K}\frac{\lambda }{\alpha^{k}}{\left|\left|W^{\left(k\right)}\right|\right|}_{1}$, subject to $\left|W_{i}^{\left(\left(1\right)\right)}\right|\geq {\left|\left|e_{i}^{\left(\left(2\right)\right)}⊙W^{\left(\left(2\right)\right)}\right|\right|}_{1}\geq \cdots \geq {\left|\left|e_{i}^{\left(\left(K\right)\right)}⊙W^{\left(\left(K\right)\right)}\right|\right|}_{1},i\in N_{d}$, where *λ* and *α* are two regularization parameters controlling the sparsity and the decay in the coefficients for interactions of different orders, *W* denotes the set of parameters {W(k)}k=1K,W((k))∈R(dk) for *k* = 1,⋯,*K* is a vector of length (dk)=d!k!(d−k)! with $W_{<i_{1},\cdots ,i_{k}>}^{\left(k\right)}$ as its element corresponding to the index <*i*_1_,⋯,*i*_*k*_>, and *L*(·) is a loss function for regression such as the square loss defined as $L(W)=1/2{\left|\left|y-\sum_{k=1}^{K}\sum_{i_{1,}\cdots ,i_{k}}^{d}W_{<i_{1},\cdots ,i_{k}>}^{\left(k\right)}z_{<i_{1},\cdots ,i_{k}>}^{\left(k\right)}\right|\right|}^{2}$ where ⊙ denotes the element-wise product of two vectors. The constraints associated with each covariate *i* have a chain of inequality constraints,which contains (*K*−1) inequality constraints and there are a total of *d* chains.

### Model comparision and parameter optimization

To make our analyses be robust, three other models were also used in comparision: the L1-norm regularized method (LASSO) [19,20], the L1- and L2-norm regularized method (Elastic Net) [21], and the L1- and L∞-norm the Composite Absolute Penalties method (iCAP) [22]. Briefly, the LASSO regression seeks to minimize the following: ||y–X • w||^2^ +λ_1_||w||1, the Elastic Net regression seeks to minimize: ||y–X • w||^2^ +λ_1_||w||1 +λ_2_||w||2, and the iCAP seeks to minimize: ||y − X • w||^2^ +λ_1_||[||wG_1_ ||γ_1_, ||wG_2_ ||γ_2_, · · ·, ||wG_n_ ||γ_n_, · · ·] ||_γ0_, where y is the vector of actual response value, w is the vector of weights, X is the matrix of explanatory value, λ_1_ is constraint parameters, and || · ||1 is the L1-norm, || · ||2 is the L2-norm, Gn’s, n = 1, · · ·, N is indices of *n*-th pre-defined group, wG_n_ is corresponding vector of weight, ||·||γ_n_ is group norm, and ||·||γ_0_ is overall norm. Here I choose γ0 equals 1 as the overall norm. If we choose that γ1 = γ2 = ··· = γN = ∞ as group norm, which we refer to as the algorithm iCAP.

To incorporate the biochemical properties of amino acids, we used three different distance measurement schemes, 1) the Protein-Protein Interations in Macromolecular Analysis (PIMA) [20,85], 2) the binary method, and 3) the 3 groups of amino acid method as described elsewhere [17] PIMA assigned 20 amino acids into nine groups and gave a different numerical coding for different mutations. Mutations between different pairs of residues were given an inclusive weight between 0 and 5. In the binary method, the element *j* (*j*th residue) of *x*_*i*_ was encoded to 1 if the residues in position *j* were different in two compared sequences and 0 if not. In the three amino acid groups method, each amino acid is assigned to one of the three groups: nonpolar (V, L, I, M, C, F, W, and Y), small nonpolar (G, A, and P), and polar/charged (S, T, N, Q, H, D, E, K, and R) based on their biophysical properties. If a specific mutation occurred between two groups in residue *j*, such as nonpolar to small polar, we encode the element *j* of *x*_*i*_ to 1; if not, we encode *j* of *x*_i_ to 0. Different from PIMA and binary scoring, we calculate bidirectional weights between two groups of amino acids. That is, the weight from nonpolar to small polar and that from small polar to nonpolar were different. With three amino acid groups, there were nine different combinations (nonpolar to small polar was different than small polar to nonpolar). In the learning results, a greater magnitude of weight indicated that the features were more significant than those with a lower magnitude of weight.

The regularization parameters in the sparse learning model were tuned based on the root mean square error (RMSE). The selection of LASSO, Elastic Net, or iCAP for the model and the selection of the scoring method were also based on the RMSE from 10-fold cross-validation, in which 90% of the data were used in training and 10% in testing. The smaller RMSE, the better the model’s performance. Our results showed that the structure-guided GHSM with PIMA, *λ* = 0.01, and *α* = 10 had the best performance and was used in our final analyses (S3 Table).

### Structural modeling and visualization

In order to model the complete three-dimensional structure of the IAV RNP complex, the reference sequence of the A/Cali/07/2009(H1N1) RNP was used in a BLAST search to find similar sequences with existing structures (https://blast.ncbi.nlm.nih.gov/). A structure of the complete A/Northern Territory/60/1968(H3N2) RNP complex without RNA (PDB ID 6QNW) and the A/duck/Fujian/01/2002(H5N1) RNP complex loaded with RNA (6QPF) were selected. To understand the functions of the residues within the context of both RNP complex and RNA, the three-dimensional PB1 structure (PDB ID 4WSB) had the RNA primer and substrate modeled into the structure by superimposing it with the reovirus polymerase structure (PDB ID 1N38) using Dali (http://ekhidna2.biocenter.helsinki.fi/dali/).

### Statistical analysis

A student’s *t* test was used to determine the statistical difference in the polymerase activities between RNPs. An alpha of 0.05 was used as the standard for significance.

## Supporting information

S1 Fig Phylogenetic analyses of RNP proteins of contemporary influenza A viruses. (A) Polymerase basic 2 protein, (B) polymerase basic 1 protein, (C) polymerase acidic protein, (D) nucleoprotein. Phylogenetic trees were inferred by using the maximum-likelihood method by running RAxML v8.2.10 with 1000 bootstrap replicates and using Gamma model rate of heterogeneity and GTR substitution model. Human, avian/avian-origin, swine, and canine strains used in this study are highlighted with magenta, yellow, red, and blue, respectively.
(TIF)

S1 Table A list of 24 double RNP reassortants among human seasonal and enzootic IAV RNP genes. (XLSX)

S2 Table A list of 17 double RNP reassortants among enzootic IAV RNP genes. (XLSX)

S3 Table Performance of sparse learning methods in feature selection. (XLSX)

S4 Table Residues in RNP proteins and their weights identified from machine learning to be associated with influenza RNP polymerase activities. The weights for both Binary and PIMA were absolute values. For three amino acid groups, A positive weight indidates a potential in enhancing polymerase activities and a negative number a potential in impairing polymerase activities.
(XLSX)
