# Supplementary figures and images for: Triple reassortment increases compatibility among viral ribonucleoprotein genes of contemporary avian and human influenza A viruses
Source: PLoS Pathog. 2021 Oct 7;17(10):e1009962. doi: 10.1371/journal.ppat.1009962 (PMC8525756; doi:10.1371/journal.ppat.1009962)

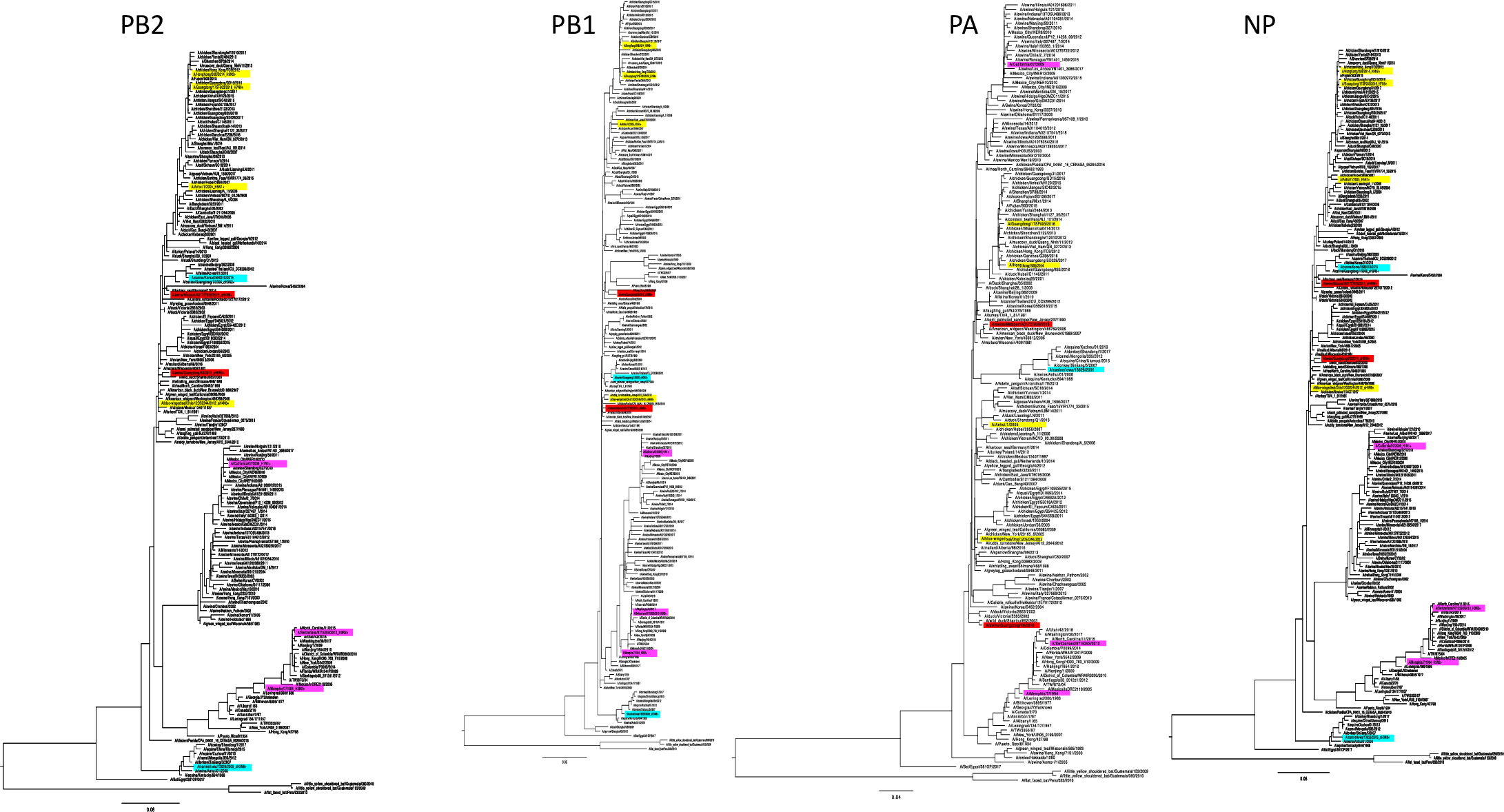

Supplement: S1 Fig — (A) Polymerase basic 2 protein, (B) polymerase basic 1 protein, (C) polymerase acidic protein, (D) nucleoprotein. Phylogenetic trees were inferred by using the maximum-likelihood method by running RAxML v8.2.10 with 1000 bootstrap replicates and using Gamma model rate of heterogeneity and GTR substitution model. Human, avian/avian-origin, swine, and canine strains used in this study are highlighted with magenta, yellow, red, and blue, respectively. (TIF) [file ppat.1009962.s001.tif]
